# A Gene Expression Atlas of the Domestic Water Buffalo (*Bubalus bubalis*)

**DOI:** 10.3389/fgene.2019.00668

**Published:** 2019-07-24

**Authors:** Rachel Young, Lucas Lefevre, Stephen J. Bush, Akshay Joshi, Salam Herojeet Singh, Santosh Kumar Jadhav, Velu Dhanikachalam, Zofia M. Lisowski, Daniela Iamartino, Kim M. Summers, John L. Williams, Alan L. Archibald, Suresh Gokhale, Satish Kumar, David A. Hume

**Affiliations:** ^1^The Roslin Institute and Royal (Dick) School of Veterinary Studies, University of Edinburgh, Edinburgh, United Kingdom; ^2^Nuffield Department of Clinical Medicine, University of Oxford, Oxford, United Kingdom; ^3^Centre for Cellular and Molecular Biology, Hyderabad, India; ^4^Central Research Station, BAIF Development Research Foundation, Pune, India; ^5^ERBAFLOR, Research and Development, Peruzzo, Basaluzzo, Italy; ^6^Mater Research Institute-University of Queensland, Translational Research Institute, Brisbane, QLD, Australia; ^7^Davies Research Centre, School of Animal and Veterinary Sciences, University of Adelaide, Adelaide, SA, Australia; ^8^School of Life Science, Central University of Haryana, Mahendergargh, India

**Keywords:** water buffalo, livestock, expression atlas, network analysis, functional annotation, FAANG

## Abstract

The domestic water buffalo (*Bubalus bubalis*) makes a major contribution to the global agricultural economy in the form of milk, meat, hides, and draught power. The global water buffalo population is predominantly found in Asia, and per head of population more people depend upon the buffalo than on any other livestock species. Despite its agricultural importance, there are comparatively fewer genomic and transcriptomic resources available for buffalo than for other livestock species. We have generated a large-scale gene expression atlas covering multiple tissue and cell types from all major organ systems collected from three breeds of riverine water buffalo (Mediterranean, Pandharpuri and Bhadawari) and used the network analysis tool Graphia Professional to identify clusters of genes with similar expression profiles. Alongside similar data, we and others have generated for ruminants as part of the Functional Annotation of Animal Genomes Consortium; this comprehensive transcriptome supports functional annotation and comparative analysis of the water buffalo genome.

## Introduction

The domestic water buffalo (*Bubalus bubalis*) has a world population of approximately 200 million^1^ distributed throughout 48 countries, making it the sixth most populous livestock species after chickens, cattle, sheep, goats, and pigs. Asia accounts for 97% of buffalo production with the largest population in India (>100 million). The water buffalo contributes significantly to global milk production, being the main milk-producing animal in India and Pakistan, as well as providing meat, hides and draught power. There are two subspecies of water buffalo, the river buffalo and swamp buffalo, which are found in separate geographical locations (Cockrill, 1981). River buffalo are widely distributed in the Indian subcontinent, the Middle East, Europe, and North Africa, whereas swamp buffalo are located in Northeast India, Bangladesh, China, and Southeast Asia. Analysis of molecular markers in river and swamp buffalo populations indicates that the subspecies were independently domesticated (Kumar et al., 2007; Lei et al., 2007; Colli et al., 2018). River buffalo have been selected for milk production. The Mediterranean breed of river buffalo produces around 2,000 kg of milk per lactation which is used in the production of dairy products such as buffalo mozzarella. Swamp buffalo have traditionally been used as draught animals, but in China and the Philippines, efforts have been made to improve dairy production by breeding them to river buffalo (Yang et al., 2013).

A draft water buffalo genome was released in 2013 and published in 2017 (Williams et al., 2017), assembled from a female Mediterranean (river) water buffalo. A new highly contiguous assembly for the river buffalo has recently been generated using long-read sequencing and other technologies (Low et al., 2019). There is no published genome sequence available for the swamp buffalo. A transcriptome of the Chinese swamp buffalo (Deng et al., 2016) was generated based upon RNA from 11 tissues collected from two Chinese swamp buffaloes (one male, one female). However, as the RNA from all tissues was pooled into a single RNA sequencing (RNA-Seq) library, this dataset provides no insight into tissue-specific expression.

Next-generation sequencing technologies allow us to generate genome-scale transcription maps providing information on both the structure and level of expression of a gene (Wang et al., 2009). The analysis of RNA-Seq data can benefit from, but is not limited by, existing knowledge of the genome, and is well suited to non-model species that lack high-quality reference genomes. RNA-Seq can be used to quantify the abundance of transcripts and identify the precise location of transcript boundaries to single base-pair resolution, depending on the technology used for library generation. Short-read sequencing technology is high-throughput and relatively cheap, and so suits the generation of a transcriptional atlas from a large-scale compendium of tissues and cell types from a given species. We previously established a transcriptional atlas for sheep (Clark et al., 2017) using this approach. We also devised a method to merge published RNA-Seq datasets from different laboratories to create an expression atlas for the chicken (Bush et al., 2018).

In the present study, we have constructed a comprehensive atlas of gene expression encompassing 220 tissue and cell samples collected from 10 river buffaloes of three different breeds (Mediterranean, Pandharpuri, and Bhadawari). We generated over 21 billion raw sequence reads which mapped to 18,730 unique genes. The dataset was used to support annotation of transcribed sequences in the new buffalo genome assembly (Low et al., 2019). Here, we use the data to analyze the patterns of expression of individual genes. These data will support functional annotation and interpretation of coding and non-coding variants associated with economically important traits and also allow comparative analysis with other ruminant and non-ruminant species.

## Materials and Methods

### Ethics Statement

Ethics approval was obtained from The Roslin Institute’s and the University of Edinburgh’s Protocols and Ethics Committees. All animal work was carried out under the regulations of the Animals (Scientific Procedures) Act 1986.

### Sample Collection and RNA Isolation

All animals used in this study were healthy. Samples were collected from six Mediterranean water buffaloes and four Indian water buffaloes (Pandharpuri and Bhadawari breeds). Tissues from the major organ systems were dissected into small pieces (100 mg) and collected into RNAlater^®^ or snap frozen in liquid nitrogen. Bone marrow, alveolar lavage, and peripheral blood mononuclear cells (PBMCs) were collected and cryopreserved at −155°C for subsequent culture and RNA extraction. Viable cell counts were performed using Trypan blue (Gibco). All cell viabilities were >90%. Bone marrow was flushed from the posterior ribs with RPMI-1640 containing 5 mM EDTA, filtered through a 100-µm cell strainer (Corning) then pelleted by centrifugation (400 × g for 5 min). Red blood cells were removed by lysis for 5 min at room temperature in RBC lysis buffer (BioLegend) then washed in phosphate buffered saline (PBS). Alveolar lavage was performed by removing the lungs and trachea, then flushing the lungs with PBS through an endotracheal tube. The lavage was then filtered through a 100-µm cell strainer (Corning) then pelleted by centrifugation (400 × g for 10 min). Alveolar macrophages were isolated from alveolar lavages by culturing them overnight in complete medium [RPMI-1640, 20% heat-inactivated fetal calf serum (FCS) (GE Healthcare), penicillin/streptomycin (Invitrogen), and GlutaMAX Supplement (Invitrogen)] supplemented with 10^4^ U/ml rhCSF1 at 10^6^ cells/ml in six-well plates. The following day, non-adherent cells were removed with the media, and remaining alveolar macrophages were collected in TRIzol (Ambion). PBMCs were isolated from whole blood by centrifuging at 1,200 × g for 15 min (no brake) to obtain buffy coats. The buffy coat was then diluted in an equal volume of PBS + 2% FCS then layered over Lymphoprep (Axis-Shield) and centrifuged at 1,200 × g for 25 min (no brake). The mononuclear cell fraction was collected and washed in PBS, then red blood cells removed by lysis as detailed above. Bone marrow-derived macrophages (BMDMs) and monocyte-derived macrophages (MDMs) were obtained by culturing bone marrow cells or PBMCs, respectively, at 10^6^ cells/ml on sterile bacteriological plastic in the presence of recombinant human colony-stimulating factor (rhCSF1; 10^4^ U/ml; gift from Chiron, Emeryville, CA) for 10–11 days. To capture inducible innate immune effector genes, BMDMs were stimulated with 100 ng/ml lipopolysaccharide (LPS) derived from *Salmonella enterica* serotype Minnesota (as described in Kapetanovic et al., 2012) and RNA extracted at 0 and 7 h.

Total RNA was isolated from 220 tissue and cell samples (**Supplementary Table 1**). RNA extractions were carried out in two laboratories (UK and India) using different extraction methods. For the Mediterranean buffalo samples, RNA was extracted using the TRIzol (Ambion) method and purified on RNeasy Mini Columns (Qiagen). Tissues were homogenized and lysed in 1 ml of TRIzol reagent using the Precellys 24 tissue homogenizing system with lysing kit CKM or CK14 depending on the tissue type, following the manufacturer’s instructions. For the Indian buffalo samples, RNA was extracted using RNAiso Plus reagent (Takara) and purified on RNeasy mini columns. Tissues were homogenized in 1 ml RNAiso Plus using a handheld homogenizer. Tissue lysates were incubated at room temperature for 5 min to allow for complete dissociation of the nucleoprotein complex. Tissues homogenized in RNAiso Plus were centrifuged at 12,000 × g for 5 min at 4°C; then, supernatants transferred to a new tube. The remaining steps were identical for both RNA extraction methods. Chloroform was added to the tissue lysate (200 µl), and tubes were shaken vigorously for 15 s then incubated for 5 min at room temperature. Samples were centrifuged for 15 min at 12,000 × g at 4°C; then, the aqueous layer containing RNA was collected and purified on RNeasy mini columns. An on-column DNase treatment was performed as per the manufacturer’s instructions. RNA concentration was measured using Qubit RNA BR Assay Kit (Thermo Fisher) and quality controlled by TapeStation using the RNA ScreenTape Kit (Agilent) to calculate the RNA integrity number (RIN). The samples taken forward for sequencing had an average RIN of 8 (minimum 6.5) and a 260/280 ratio of 2.

### Library Preparation and Sequencing

Illumina TruSeq Stranded Total and mRNA libraries were generated and sequenced by Edinburgh Genomics using the Illumina TruSeq Stranded library protocols for total RNA library preparation (part: 15031048, revision E) and mRNA library preparation (part: 15031047, revision E). Briefly, ribosomal RNA (rRNA) was depleted from samples for total RNA-Seq, using biotinylated, target-specific oligonucleotides with Ribo-Zero rRNA removal beads. Following purification, the RNA was fragmented and first-strand cDNA synthesis performed. The RNA template was then removed, and a replacement strand was synthesized incorporating dUTP in place of dTTP to generate double-stranded (ds) cDNA. The incorporation of dUTP quenches the second strand during the subsequent PCR amplification step as the polymerase will not incorporate past this nucleotide. The ds cDNA was purified; then, the 3’ ends adenylated, and indexing adapters ligated to both ends before PCR enrichment of the library. For the TruSeq Stranded mRNA libraries, poly-A-containing mRNA was purified from total RNA using poly-T oligos attached to magnetic beads. From this point, the mRNA library protocol did not differ from the protocol for total RNA library preparation. The libraries were quality controlled using an Agilent Bioanalyzer DNA 1000 Chip and quantified by qPCR before hybridization onto a flow cell. TruSeq Stranded total RNA-Seq and mRNA-Seq libraries were sequenced using an Illumina HiSeq 2500 sequencer at depths of >100 million and >25 million 125-bp paired-end reads per sample, respectively.

### Expression-Level Quantification

The sequence data for the buffalo atlas were processed using two different methods, one alignment-free and one alignment-based, as described in Clark et al. (2017). All expression-level estimates for the atlas, expressed as transcripts per million (TPM), were obtained using the high-speed transcript quantification tool Kallisto (Bray et al., 2016), which is an alignment-free method. Kallisto creates an index of k-mers from a set of reference transcripts and then uses the k-mers of each read to “pseudoalign” each read to this index. This method assigns reads to their transcript of origin without the time-consuming step of base-level alignment. Expression levels are then estimated, per transcript, as a function of the assigned reads, with transcript-level estimates summarized to the gene-level. The accuracy of Kallisto’s estimates therefore depends on the quality of the reference transcripts, and by extension, the k-mers derived from them. As the water buffalo genome at the time of creating the index had a comparatively fragmented assembly and an incomplete transcriptomic catalogue, we used an additional, alignment-based method to identify transcript models not initially available for use by Kallisto. This second method, employing the HISAT aligner (Kim et al., 2015) and StringTie assembler (Pertea et al., 2015), was used to identify novel gene and transcript models, both protein-coding and non-coding (described below).

Using both methods together, we progressively revised the Kallisto index and updated expression-level estimates accordingly. This iterative “multi-pass” approach to Kallisto has been described previously (Clark et al., 2017) and used to create comparable gene-level expression estimates from the otherwise distinct mRNA-Seq and total RNA-Seq libraries for the sheep expression atlas (Clark et al., 2017).

For the “first pass,” we ran Kallisto on all samples, using as its index the complete set of 45,402 predicted transcripts for the draft *B. bubalis* assembly UMD_CASPUR_WB_2.0 (ftp://ftp.ncbi.nlm.nih.gov/genomes/all/GCF/000/471/725/GCF_000471725.1_UMD_CASPUR_WB_2.0/GCF_000471725.1_UMD_CASPUR_WB_2.0_rna.fna.gz, downloaded 22nd April 2016). To aid in the presentation of findings, standardized placeholder IDs were—if unavailable—assigned to each gene, transcript, and exon model. These IDs are assigned arbitrarily and are of the form geneX, rnaY, and exonZ, respectively, where X, Y, and Z are numeric. IDs are available *via* the University of Edinburgh DataShare portal (http://dx.doi.org/10.7488/ds/2292).

We then parsed these “first pass” data, which comprised of approximately 22 billion pseudoalignments (**Supplementary Table 2**), capturing 94% of the known (UMD_CASPUR_WB_2.0) genes (**Supplementary Table 3**), to revise the Kallisto index. This revision was undertaken in order to include, in the second index, those transcripts that had been erroneously omitted (i.e., where the reference annotation was incomplete), and to exclude those transcripts that had been erroneously included (i.e., spurious models due to the comparatively poor assembly). For the first criterion, we obtained the subset of reads that Kallisto did not align and assembled these *de novo* into putative transcripts. Transcripts were retained only if they showed coding potential (using the online tool CPAT v1.2.2; Wang et al., 2013) and encoded a protein similar to one of known function (**Supplementary Table 4**). This annotation process is more fully detailed in (Clark et al., 2017). After the “first pass,” we also identified and discarded those members of the reference transcriptome for which TPM was 0 in all samples. Two thousand three hundred and three transcripts were also removed from the original index because they were low-quality predictions: the RefSeq transcript required modification relative to its underlying genome sequence to create a complete CDS. Kallisto was then re-run on all samples using this revised index.

This “two pass” method was previously used to create an expression atlas for the domestic sheep (Clark et al., 2017). We also reconstructed novel transcript models for the buffalo using an alignment-based approach to process RNA-Seq data which combined the HISAT aligner with the StringTie assembler. The new transcript models created (732 protein-coding transcripts, representing 631 genes, plus 6,756 lncRNAs) were then integrated with the “second pass” Kallisto index to create a “third pass” index, with expression quantification repeated as above. Transcript models were retained only if they could be robustly annotated, using the criteria described in Clark et al. (2017) for protein-coding genes, and the criteria described in Bush et al. (2018) for lncRNAs.

The StringTie assembly is accurate with respect to the draft annotation, reconstructing all existing exon models and 82% of the transcript models (**Supplementary Table 5**). None of the gene models are precisely reconstructed because the existing draft annotation considers “gene start” and “gene end” coordinates to be the start of the first, and end of the last, CDS, respectively, irrespective of 5’ and 3’ untranslated regions (UTRs).

In the transcriptome assembly created here, thousands of new transcript models are predicted, although in the absence of experimental verification, it is not easy to determine which are plausible, as opposed to stochastic noise in RNA processing or assembly artifacts. A large number of false positive transcripts are expected as the assembly integrates both mRNA-Seq and total RNA-Seq datasets. The latter measures nascent (ongoing) transcription (Ameur et al., 2011) and consequently has a larger proportion of retained introns arising from incompletely spliced pre-mature (nuclear) mRNA (Zhang et al., 2015), which can lead to transcript assembly artifacts.

Novel transcript models were retained only if they could be robustly annotated as protein-coding. To do so, the longest ORF in each exon of its set of exon models was identified. To include this transcript in the “third pass” index, we required that a) for every exon, the longest ORF is on the same strand; b) the last ORF terminates in a stop codon, rather than simply because the ORF remains open until the end of the exon; c) although the ORF of every internal exon does not have to span the entire exon length (because there may be noise in the placement of the exon/intron boundary), no internal ORF contains a stop codon (i.e., the ORF must end when the exon does); and d) the peptide, concatenated from the set of translated ORFs, is ≥50 amino acids in length. These peptides were then aligned against the NCBI non-redundant (nr) peptide database v77 (Pruitt et al., 2005) using blastp with a scoring threshold of *p* ≤ 1e-25 (Altschul et al., 1997).

Conservative criteria were applied to parse these alignments. For a novel transcript model to be retained, ≥ 5 alignments were required, at least one of which is to a gene model from a ruminant genus [*Bison* (bison), *Bos* (cow, yak), *Camelus* (camel), *Capra* (goat), *Ovis* (sheep, mouflon), *Pantholops* (antelope), or *Vicugna* (alpaca) sources are listed in **Supplementary Table 6**]. Each alignment had to a) have a % identity within the aligned region of ≥90%, b) have an alignment length ≥ 90% of the length of the query protein, c) have an alignment length ≥ 50 amino acids, d) have no gaps, and e) not be a protein labeled “low quality,” “hypothetical,” “unnamed,” “uncharacterized” or “putative,” or otherwise have only a third-party annotation (as these can be by inference, not experiment). The set of novel transcript models derived from the StringTie assembly, after applying these filter criteria, is summarized in **Supplementary Table 7**. Their expression across the atlas is detailed in **Supplementary Table 10**, and the number of tissues with detectable expression (TPM > 1) quantified in column H “Expression summary” A GTF file containing the new gene models is available for download through the University of Edinburgh DataShare portal (http://hdl.handle.net/10283/3356).

Using this “third pass” index, on average 60–70% of the known buffalo (UMD_CASPUR_WB_2.0) protein-coding genes were detectably expressed (average TPM, across all replicates, >1) in all tissues (**Supplementary Table 8**).

### Data Downloaded From Public Repositories

To supplement the data generated herein, we integrated additional buffalo transcriptome data from the European Nucleotide Archive (ENA) under accession number PRJEB4351. These data were generated to provide reference RNA-Seq data as part of the International Water Buffalo Genome Project (Williams et al., 2017). The data comprise 30 tissues collected from a male and a female Mediterranean buffalo. Details of the tissue samples included from this project are provided in **Supplementary Table 9**.

### Network Analysis

Expression data were represented as average transcripts per million (TPM) per gene per tissue. To visualize the data, we used the network analysis tool Graphia Professional^2^ (formerly Miru, derived from BioLayout *Express* ^3D^; Freeman et al., 2007; Theocharidis et al., 2009) to create a gene-to-gene pairwise Pearson correlation matrix across all samples. To remove noise, we restricted analysis to those genes with average TPM >10 in at least one tissue. Retaining only correlations of *r* ≥ 0.80, a gene-to-gene network graph was constructed connecting 15,752 nodes (genes) with 1,851,403 edges (correlations between nodes). The Markov cluster algorithm (MCL) (van Dongen and Abreu-Goodger, 2012) was used with an inflation value (which determines cluster granularity) of 2.2 to identify clusters of co-expressed genes. Clusters are numbered according to their relative size, the largest cluster being cluster 1, and so on. The contents of the largest 50 clusters and gene expression compared to that of other species using BioGPS^3^ were examined (Wu et al., 2009; Wu et al., 2013; Wu et al., 2016). Clusters were characterized by their tissue-specificity or biological process. In cases where unannotated genes were co-expressed with annotated genes, this information could be used to reinforce suggested annotations based on conservation of synteny and sequence similarity. Gene ontology enrichment analysis of clusters was performed using PANTHER^4^. PANTHER is a classification system comprised of tools to analyze large-scale genome-wide data for gene function and pathway information (Mi et al., 2013).

### Data Availability

Sample metadata for all tissue and cell samples, prepared in accordance with Functional Annotation of Animal Genomes (FAANG) Consortium metadata standards (Harrison et al., 2018), were deposited in the EBI BioSamples database^5^ under project identifier GSB-5402 (https://www.ebi.ac.uk/biosamples/samples/SAMEG326824). RNA-Sequencing data were deposited in the European Nucleotide Archive (ENA)^6^ under accession PRJEB25226 (https://www.ebi.ac.uk/ena/data/view/PRJEB25226). All experimental protocols are available on the FAANG consortium website^7^ at http://ftp.faang.ebi.ac.uk/ftp/protocols.

The complete “third pass” expression atlas, including samples derived from (Williams et al., 2017), is available as (**Supplementary Table 10**).

## Results and Discussion

### Generating the Gene Expression Atlas

The core of this dataset was derived from four 6-month old Mediterranean buffalo. From these animals, we collected tissues from all major organ systems and, wherever possible, collected biological replicates from each sex. These tissue samples were supplemented with immune cells from two additional animals of the same breed. Collectively, the Mediterranean buffalo contributed 164 samples to the atlas. We also collected the same set of tissues from our Indian buffalo cohorts which, due to restricted availability, were older (5–7 years old). Biological replicates (2 males, 2 females) were collected where possible. The Indian animals contributed 56 samples to the atlas. A number of immune cell types were sampled, including different subsets of macrophages and their progenitors (alveolar macrophages, MDMs, BMDMs +/− LPS, bone marrow cells, and PBMCs). Previous projects in several species have indicated that macrophages are a rich source of novel mRNAs (Carninci et al., 2005; Clark et al., 2017). A complete list of the tissues sequenced can be found in **Supplementary Table 1**.

Two types of library were generated to capture the expression of the largest diversity of RNA species possible, ribo-depleted total RNA, and (polyA) mRNA. These two library types were sequenced at different depths: total RNA at >100 million paired-end read depth and mRNA at >25 million paired-end read depth, generating approximately 21 billion raw reads in total.

We selected a wide range of tissues for the atlas to obtain the largest diversity of transcripts possible, in addition to integrating 30 RNA-Seq libraries from a previous study (detailed in **Supplementary Table 9**). The final expression atlas (**Supplementary Table 10**) was the product of a three-step approach used to iteratively improve the reference transcriptome; it contains 21,537 genes expressed in at least one tissue in the buffalo atlas (**Supplementary Table 3**). The proportion of protein-coding genes from this annotation detected in each tissue is summarized in **Supplementary Table 8**. Over 93% of protein-coding genes were expressed in at least one replicate of each tissue in the atlas alongside approximately 99% of the remaining (primarily RNA) genes (**Supplementary Table 3**).

### Visualizing the Data

**Figure 1:**
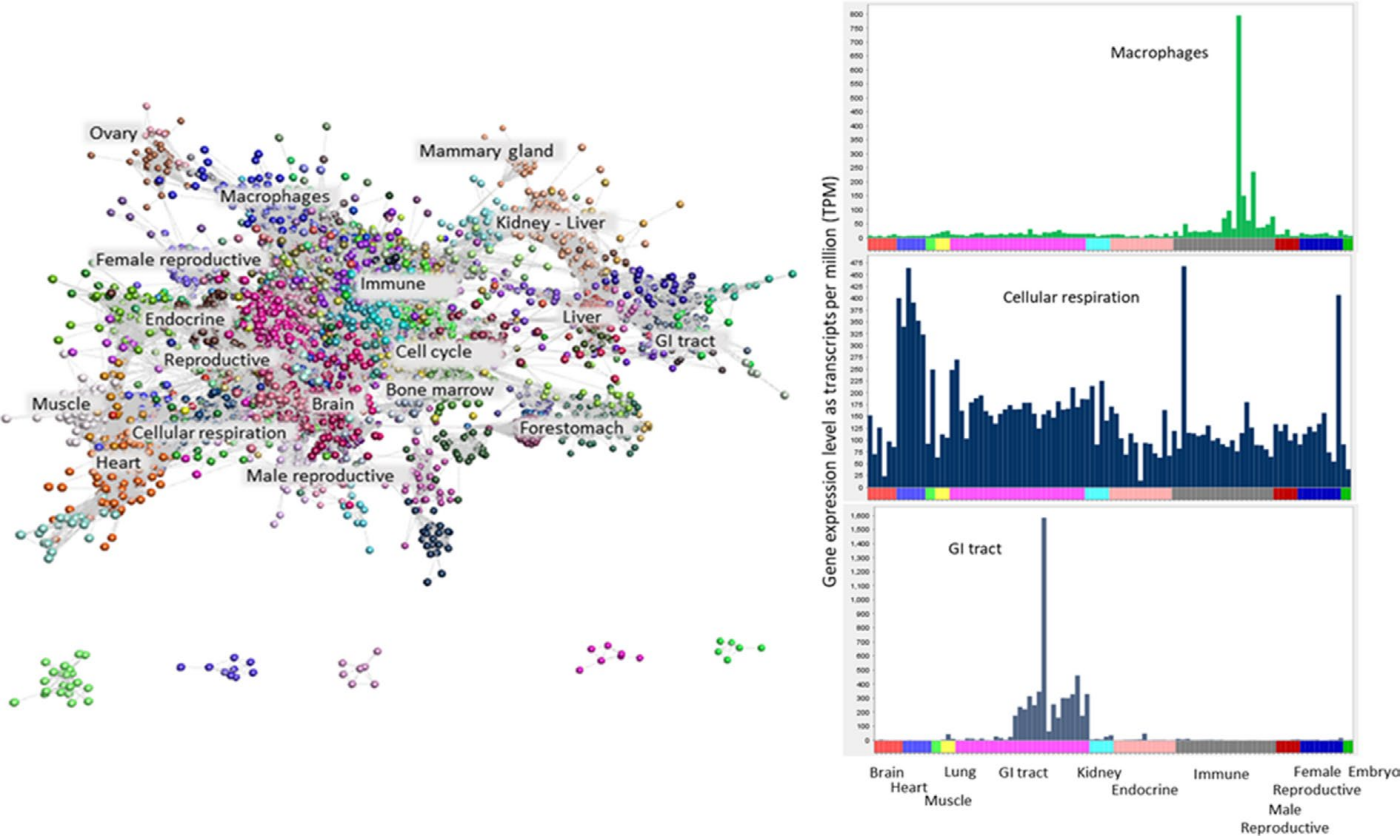
Clustered network graph of the buffalo transcriptome. The buffalo atlas data were visualised by a network graph based on Pearson correlation co-efficients for gene expression patterns. Each node represents a transcript and each edge (line) represents the correlation between individual measurements above a threshold of r = 0.80. The graph comprises 15,752 nodes connected by 1,851,403 edges. Clustering of the graph using the MCL algorithm was used to assign genes to classes or clusters based on their co-expression. The clusters can be annotated depending on the tissue specificity or cellular process by function of their contents. Plots of average expression profile for a few selected clusters are given on the right. The samples used to generate the graph are shown on the X axis ordered by organ system (colour-coded) and the Y axis shows the average TPM for the cluster. The tissue specificity of gene co-expression in three selected clusters are shown. These clusters contain genes that are highly expressed in macrophages, cellular respiration and the gastrointestinal tract.

Methods such as weighted correlation network analysis (WGCNA) and partial correlation and information theory (PCIT) have been used by others to perform gene co-expression analysis in livestock species (Watson-Haigh et al., 2010; Alexandre et al., 2015; Weber et al., 2016; Salleh et al., 2018). We have chosen to use Graphia Professional, an alternative tool for the visualization and analysis of network graphs from large RNA-Seq and microarray datasets (Freeman et al., 2012; Mabbott et al., 2013; Clark et al., 2017). Graphia filters out genes with low expression and stably expressed genes and thus highlights the most variable, likely tissue-specific genes. A gene-to-gene correlation matrix for the buffalo atlas was calculated, and a weighted network graph constructed using a Pearson correlation of *r* ≥ 0.8 (see Materials and Methods). As we have done for atlas projects on other species (Clark et al., 2017; Bush et al., 2018; Freeman et al., 2012; Mabbott et al., 2013), the correlation threshold was determined empirically using a functionality within Graphia to maximize the number of nodes (genes) included whilst minimizing the number of edges. The optimal threshold is similar to previous projects and has been validated by the unequivocal GO term enrichment in specific clusters. The resulting graph contained 15,752 nodes (genes) connected by 1,851,403 edges and was clustered using the Markov clustering algorithm (MCL) at an inflation value of 2.2. Clusters with fewer than five nodes were excluded from further analysis, resulting in 276 clusters ranging from 5 to 3,372 nodes. The network graph is shown in **Figure 1**, along with the expression profiles of selected clusters. The graph consisted of one large component containing 12,993 nodes and 1,807,061 edges, and five smaller components each containing ≤21 nodes.

The content of the top 50 clusters is summarized in **Table 1**, in which clusters are labeled according to the tissue or cell type showing highest expression in the cluster. The full list of clusters and their contents are available in **Supplementary Table 11**. As noted in several other atlas projects (Freeman et al., 2012; Mabbott et al., 2013; Clark et al., 2017; Bush et al., 2018), the largest cluster (cluster 1) consisted mainly of housekeeping genes, with expression detected in all tissues in the atlas. This cluster contained many transcripts that lack informative annotation, reflecting the focus within the literature on tissue-specific gene expression and on genes in which genetic variation is associated with a phenotype other than lethality. Another large cluster (cluster 3) contained 636 genes, around 80% of which are annotated. These genes showed peaks of expression in PBMCs, spleen, and endometrium but were otherwise also widely expressed across diverse tissues. Genes expressed in this cluster were enriched for GO terms including “macromolecule metabolic process” (GO ID: 0043170, *p* = 6.39 × 10^−22^), “regulation of RNA metabolic processes” (GO ID: 0051252, *p* = 9.43 × 10^−18^), and “regulation of gene expression” (GO ID: 0010468, *p* = 9.95 × 10^−18^) (**Table 1**). As the enriched biological processes suggest, several of the genes present in this cluster are involved in gene regulation, including genes encoding transcription factors such as various zinc finger proteins along with argonaute 2 (*AGO2*), the catalytic component of the RNA-induced silencing complex (RISC), and casein kinase 1 alpha 1 (*CSNK1A1*), the inhibitory kinase of AGO2. CSNK1A1 initiates the AGO2 phosphorylation cycle, required to enhance the specificity of miRNAs for their target (Golden et al., 2017).

**Table 1:** Fifty largest clusters from network analysis.

Cluster ID	No. of transcripts	Tissue specificity	Class	Biological process	GO term	*p*-value
1	3,372	Endometrium > spleen > general, relatively even	Housekeeping	Cellular nitrogen compound metabolic process	GO:0034641	1.91 × 10^−56^
2	648	Oviduct > fallopian tube > testis > endometrium	Reproductive system	Cilium organization Cilium assembly	GO:0044782 GO:0060271	6.67 × 10^−29^ 9.07 × 10^−29^
				Cell projection assembly	GO:0030031	1.04 × 10^−26^
3	636	PBMCs > endometrium > spleen > general	Housekeeping	Macromolecule metabolic process	GO:0043170	6.39 × 10^−22^
4	542	Testis (Bhadawari)	Male reproductive	Male gamete generation	GO:0048232	7.50 × 10^−14^
				Spermatogenesis	GO:0007283	4.76 × 10^−13^
5	381	Cerebellum > hippocampus	CNS	nervous system development	GO:0007399	2.50 × 10^−20^
				generation of neurons	GO:0048699	2.82×10^−17^
6	337	Embryo	Developmental	No statistically significant results		
7	314	White blood cells (WBC)	Immune	Response to cytokines	GO:0034097	1.42 × 10^−8^
				Immune system process	GO:0002376	1.43 × 10^−8^
				Regulation of immune system process	GO:0002682	5.47 × 10^−7^
8	312	Spleen > WBC > PBMCs > lymph nodes > immune tissues	Immune	Immune system process	GO:0002376	1.25 × 10^−18^
				Regulation of immune system process	GO:0002682	6.63 × 10^−13^
9	245	General	Housekeeping	No statistically significant results		
10	227	Peyer’s patches > ileum > bone marrow > thymus > spleen	Pathway	Cell cycle Mitotic cell cycle	GO:0007049 GO:0000278	1.14 × 10^−57^ 1.80 × 10^−44^
				Cell cycle process	GO:0022402	5.60 × 10^−42^
11	225	Spinal cord > obex > hippocampus > cerebellum	CNS	Axon ensheathment Glial cell differentiation	GO:0008366 GO:0007272	6.92 × 10^−10^ 1.38 × 10^−9^
				Ensheathment of neurons	GO:0042552	9.8 × 10^−9^
12	192	Heart > left ventricle > right ventricle > right atrium > thoracic esophagus	Cardiovascular system	Muscle structure development Regulation of heart contraction	GO:0061061 GO:0008016	4.59 × 10^−8^ 1.19 × 10^−6^
				Striated muscle tissue development	GO:0006941	1.67 × 10^−6^
13	191	Kidney cortex > kidney medulla > liver	Renal/endocrine system	Organic acid metabolic process Small molecule metabolic process	GO:0006082 GO:0044281	3.23 × 10^−19^ 4.81 × 10^−18^
				Carboxylic acid metabolic process	GO:0019752	1.60 × 10^−17^
14	178	Semitendinosus muscle > thoracic esophagus > longitudinal muscle > tongue	Muscular system	Muscle system process Muscle structure development Striated muscle cell differentiation	GO:0003012 GO:0061061 GO:0051146	1.44 × 10^−17^ 2.51 × 10^−16^ 8.85 × 10^−14^
15	157	Liver	Liver	Blood coagulation	GO:0007596	2.09 × 10^−18^
				Hemostasis	GO:0007599	3.13 × 10^−18^
				Coagulation	GO:0050817	6.26 × 10^−18^
16	155	Peyer’s patches > ileum > bone marrow > thymus > spleen	Immune	DNA metabolic process	GO:0006259	1.07 × 10^−7^
17	153	Endometrium > spleen > heart > general	Pathway	Cellular respiration	GO:0045333 GO:0055114	1.29 × 10^−44^ 6.62 × 10^−43^
				Energy derivation by oxidation of organic compounds	GO:0015980	8.91 × 10^−41^
18	152	Endometrium > embryo > spleen > general	Housekeeping	Macromolecule metabolic process Gene expression	GO:0043170 GO:0010467	4.42 × 10^−6^ 1.28 × 10^−5^
				Cellular macromolecule metabolic process	GO:0044260	1.53 × 10^−5^
19	148	Alveolar macrophages > BMDM + LPS	Immune	Immune system process Regulation of cytokine production	GO:0002376 GO:0001817	2.12 × 10^−11^ 2.60 × 10^−11^
				Positive regulation of defense response	GO:0031349	1.63 × 10^−10^
20	135	Macrophages > spleen	Immune	Collagen catabolic process	GO:0030574	5 × 10^−5^
				Regulation of immune system process	GO:0002682	6.67 × 10^−5^
				Collagen metabolic process	GO:0032963	1.20 × 10^−3^
21	131	Omasum > rumen > reticulum > abomasum > tongue > tonsil	GI tract	Epidermis development Skin development	GO:0008544 GO:0043588	3.48 × 10^−10^ 9.26 × 10^−8^
				Epidermal cell differentiation	GO:0009913	3.32 × 10^−7^
22	112	Spleen > lymph nodes > small and large intestine > lung	Immune	Immune system process Immune response	GO:0002376 GO:0006955	8.51 × 10^−10^ 1.39 × 10^−5^
				Regulation of immune system process	GO:0002682	1.27 × 10^−4^
23	111	Bone marrow > spleen > BMDM +/− LPS	Immune	Protoporphyrinogen IX metabolic process	GO:0046501	6.87 × 10^−5^
				Porphyrin-containing compound metabolic process	GO:0006778	9.05 × 10^−5^
				Tetrapyrrole metabolic process	GO:0033013	9.17 × 10^−5^
24	105	Endometrium > oviduct > fallopian tube > testis > epididymis > general	Reproductive system	Cilium organization Cilium assembly Cell projection assembly	GO:0044782 GO:0060271 GO:0030031	2.79 × 10^−7^ 2.59 × 10^−5^ 1.02 × 10^−4^
25	102	Endometrium > cerebellum > spinal cord > obex	CNS	Ion transmembrane transport Regulation of membrane potential Regulation of trans-synaptic signaling	GO:0034220 GO:0042391 GO:0099177	2.27 × 10^−2^ 2.45 × 10^−2^ 2.52 × 10^−2^
26	102	Small and large intestine	GI tract	Brush border assembly	GO:1904970	2.25 × 10^−4^
				Regulation of microvillus organization	GO:0032530	1.02 × 10^−2^
				Regulation of cell projection size	GO:0032536	1.45 × 10^−2^
27	100	Omasum > rumen > reticulum > rectum > abomasum > cecum	GI tract	Supramolecular fiber organization Actin filament organization	GO:0097435 GO:0007015	3.74 × 10^−2^ 4.25 × 10^−2^
				Actin crosslink formation	GO:0051764	5.85 × 10^−2^
28	90	Epididymis > testis	Male reproductive system	No statistically significant results		
29	79	Ovary follicle > ovary	Female reproductive system	Regulation of hormone levels Sulfur compound metabolic process Chondroitin sulfate metabolic process	GO:0010817 GO:0006790 GO:0030204	6.43 × 10^−4^ 7.75 × 10^−4^ 9.75 × 10^−4^
30	76	Pituitary gland > endometrium	Endocrine system	Endocrine system development	GO:0035270	3.19 × 10^−8^
				Pituitary gland development	GO:0021983	1.7 × 10^−7^
				Diencephalon development	GO:0021536	9.21 × 10^−7^
31	73	Kidney cortex > kidney medulla	Renal system	Transmembrane transport	GO:0055085	7.50 × 10^−5^
				Ion transport	GO:0006811	1.11 × 10^−4^
				Inorganic anion transport	GO:0015698	1.86 × 10^−4^
32	72	Adrenal gland	Endocrine system	Organic hydroxy compound transport	GO:0015850	6.24 × 10^−5^
				Monoamine transport	GO:0015844	1.1 × 10^−4^
				Serotonin uptake	GO:0051610	2.92 × 10^−3^
33	62	Tongue > rumen > reticulum > tonsil	GI tract	No statistically significant results		
34	59	General	Housekeeping	No statistically significant results		
35	54	Testis > Peyer’s patches > ileum	Pathway	Nuclear transport	GO:0051169	1.35 × 10^−2^
				Protein-containing complex localization	GO:0031503	1.94 × 10^−2^
				Nucleocytoplasmic transport	GO:0006913	2.03 × 10^−2^
36	49	Embryo	Developmental	No statistically significant results		
37	48	Thyroid	Endocrine system	No statistically significant results		
38	48	General	Pathway	Amide transport	GO:0042886	8.81 × 10^−6^
				Protein transport	GO:0015031	8.92 × 10^−6^
				Peptide transport	GO:0015833	8.94 × 10^−6^
39	47	Ovary	Endocrine system	Not statistically significant		
40	45	Thyroid > salivary gland > kidney medulla > lung	Endocrine system	Glycoprotein metabolic process One-carbon metabolic process	GO:0009100 GO:0006730	4.13 × 10^−2^ 6.09 × 10^−2^
41	43	Endometrium > epididymis > testis > fallopian tube > ovary follicle	Reproductive system	Regulation of animal organ morphogenesis Heart morphogenesis	GO:2000027 GO:0003007	2.29 × 10^−2^ 3.1 × 10^−2^
				Heart development	GO:0007507	3.50 × 10^−2^
42	41	Thoracic esophagus > tongue > semitendinous muscle > left ventricle	Musculoskeletal system	No statistically significant results		
43	41	General	Pathway	Protein folding	GO:0006457	2.66 × 10^−12^
				Positive regulation of protein localization to chromosome, telomeric region	GO:1904816	6.19 × 10^−12^
				Regulation of establishment of protein localization to chromosome	GO:0070202	6.31 × 10^−12^
44	41	White blood cells > endometrium > spleen	Pathway	Protein modification process Macromolecule modification	GO:0036211 GO:0043412	7.93 × 10^−3^ 8.55 × 10^−3^
				Cellular protein modification process	GO:0006464	1.19 × 10^−2^
45	37	Occipital lobe > hippocampus	CNS	No statistically significant results		
46	36	Lung > lymph nodes > epididymis	No class	Cardiovascular system development Vasculature development	GO:0072358 GO:0001944	1.12 × 10^−11^ 1.35 × 10^−11^
				Blood vessel development	GO:0001568	1.42 × 10^−11^
47	34	Mammary gland	Reproductive system	Proximal/distal pattern formation involved in nephron development	GO:0072047	1.71 × 10^−2^
				Specification of loop of Henle identity	GO:0072086	1.90 × 10^−2^
				Pattern specification involved in kidney development	GO:0061004	1.99 × 10^−2^
48	33	General	No class	No statistically significant results		
49	33	Lymph nodes > lung	Immune	Synapse pruning	GO:0098883	1.78 × 10^−4^
				Innate immune response	GO:0045087	1.34 × 10^−3^
				Lymphocyte-mediated immunity	GO:0002449	3.09 × 10^−2^
50	31	Lymph nodes > small and large intestine	Immune	Immune system process Immune system development	GO:0002376 GO:0002520	5.87 × 10^−8^ 1.40 × 10^−5^
				Leukocyte activation	GO:0045321	5.26 × 10^−5^

A list of the 50 largest co-expression clusters from the water buffalo gene expression atlas. Clusters are numbered according to their size (the largest is cluster 1). The first two columns give the cluster ID and number of transcripts present in that cluster, the following two columns describe tissue specificity and class (where possible) and the final three columns describe the biological process of the genes co-expressed in that cluster, their GO term and the associated p-value cluster according to gene ontology enrichment analysis using PANTHER.

Most of the smaller clusters contained genes whose expression was restricted to an organ system. Some clusters were specific to a single tissue or cell type, while others were clearly associated with a biological or cellular process. In many cases, the likely function of genes within any of the clusters can be inferred from their cell type enrichment or the known function of well-annotated genes within the clusters. These include organ system and tissue-specific clusters for genes expressed predominantly in the brain (clusters 5, 11, 25, and 45), heart (clusters 12 and 66), and reproductive system (clusters 2, 4, 24, 28, 29, 39, and 41). More specifically, certain clusters were enriched for the biological process GO terms of cilium organization (*p* = 6.67 × 10^−29^) and cilium assembly (*p* = 9.07 × 10^−29^) (cluster 2), male gamete generation (*p* = 7.50 × 10^−14^) and spermatogenesis (*p* = 7.50 × 10^−14^) (cluster 4), nervous system development (*p* = 2.50 × 10^−20^) and generation of neurons (*p* = 2.82 × 10^−17^) (cluster 5), and muscle structure development (*p* = 4.59 × 10^−8^) and regulation of heart contraction (*p* = 1.19 × 10^−6^) (cluster 12).

We noted that replicate samples sometimes showed different expression patterns. For example, the expression of some genes in the three testis samples were not consistent. These differences may result from the differing ages of the animals (from 6 months to more than 5 years). Variation in other tissues may result from sex-specific effects, phase of oestrus cycle in the females, different husbandry (for example diet, exercise level, ambient temperature), and other factors. These differences could be explored further with a larger set of replicates. Nevertheless, in this analysis, clear, logical associations of gene expression patterns were found in spite of some differences between replicates, as presented below.

### Immune System Clusters

We sampled several immune tissue and cell populations to identify genes that might be associated with disease resistance and resilience traits. We identified two main macrophage clusters (clusters 19 and 20) from the atlas data, each enriched for a particular macrophage subset. Genes in cluster 19 showed the highest expression levels in alveolar macrophages (AM) with many of the genes encoding well characterized macrophage-specific proteins. Genes in this cluster were enriched for GO terms including “immune system process” (*p* = 2.12 × 10^−11^), “regulation of cytokine production” (*p* = 2.60 × 10^−11^), and “positive regulation of defense response” (*p* = 1.63 × 10^−10^). Genes in this cluster include those encoding pro-inflammatory cytokines IL1A, IL1B, IL6, and IL8 and toll-like receptors TLR2 and TLR4 and the arginine metabolizing enzymes arginase (ARG2) and nitric oxide synthase (NOS2). The expression of *ARG2* and *NOS2* differs in buffalo macrophages from human, mouse, pig, and sheep and is more similar to cattle gene expression as previously described (Young et al., 2018). A separate macrophage-specific cluster (cluster 20) showed peak expression in MDMs and contained the macrophage-expressed genes *CD14*, *CD63*, and *CD68*; cytokine receptor genes *CCR1* and *CCR5*; and myeloid cell marker gene *TREM2*. Most of these genes were detected in a macrophage cluster of the pig atlas (Freeman et al., 2012). In sheep as in buffalo, *TREM2* is expressed in MDMs and LPS-stimulated macrophages and has very low expression in AMs (Clark et al., 2017). Genes in this cluster were also enriched for the GO terms “collagen catabolic process” (*p* = 5 × 10^−5^), “regulation of immune system process” (*p* = 6.67 × 10^−5^), and “collagen metabolic process” (*p* = 1.20 × 10^−3^).

Cluster 23 contains genes with expression peaks in bone marrow and spleen and both LPS-stimulated and unstimulated BMDMs. Biological process GO terms enriched in this cluster include “protoporphyrinogen IX metabolic process” (*p* = 6.87 × 10^−5^), “porphyrin-containing compound metabolic process” (*p* = 9.05 × 10^−5^), and “tetrapyrrole metabolic process” (*p* = 9.17 × 10^−5^). Genes in this cluster include those encoding some of the key red blood cell transcription factors, GATA1, GFI1B, and KLF1. Genes for members of the heme biosynthesis pathway were also expressed in this cluster, including *ALAS2*, *FECH*, *HMBS*, *UROD*, and *UROS*. In addition, the solute carrier genes *SLC4A1* and *SLCO4C1* were predominantly expressed in buffalo bone marrow cells. By inference, many of the genes within these clusters that currently lack a functional annotation are likely to have an immune function.

### Cellular Processes

As previously observed in both the sheep and pig expression atlases (Freeman et al., 2012; Clark et al., 2017), genes involved in different biological processes may be active in many cells or tissues, and so clusters enriched for these processes can be identified. Genes involved in cellular respiration (glycolysis, the TCA cycle, and oxidative phosphorylation) clustered together in buffalo cluster 17. In the pig atlas (Freeman et al., 2012), components of the oxidative phosphorylation complex and related pathways encoded by the nuclear genome clustered together and showed elevated expression in the heart. The equivalent genes encoded by the mitochondrial genome clustered separately in pigs. In buffalo, several genes involved in the TCA cycle were present in cluster 17 along with components of all five oxidative phosphorylation complexes associated with ATP generation in the mitochondria (summarized in **Table 2**). This cluster contained 153 genes, most of which are involved in ATP generation. Genes in this cluster were expressed in most tissues in the atlas but clustered together because of shared high expression in the heart, cerebellum, and spleen. There were 26 unannotated genes in cluster 17, which by association are also likely to be involved in cellular respiration.

**Table 2:** Genes associated with oxidative phosphorylation (cluster 17).

Associated pathway	Genes
TCA cycle	*ACADVL, ACO2, CS, DLAT, DLST, ESRRA, FH, IARS2, IDH2, IDH3B, IDH3G, MDH1, MDH2, MPC2, PDHB, SUCLA2, SUCLG1*
Oxidative phosphorylation complex I	*NDUFA1, NDUFA2, NDUFA3, NDUFA5, NDUFA7, NDUFA8, NDUFA9, NDUFA10, NDUFA11, NDUFA12, NDUFA13, NDUFAB1, NDUFB3, NDUFB4, NDUFB5, NDUFB6, NDUFB7, NDUFB8, NDUFB9, NDUFB10, NDUFB11, NDUFC1, NDUFC2, NDUFS1, NDUFS2, NDUFS3, NDUFS5, NDUFS6, NDUFS7, NDUFS8, NDUFV1, NDUFV2*
Oxidative phosphorylation complex II	*SDHA, SDHB, SDHC, SDHD*
Oxidative phosphorylation complex III	*TUFM, UQCRFS1*
Oxidative phosphorylation complex V	*ATP5A1, ATP5B, ATP5C1, ATP5D, ATP5E, ATP5F1, ATP5G1, ATP5G3, ATP5H, ATP5J, ATP5J2, ATP5L, ATP5O, ATP5SL, ATPIF1*
Mitochondrial membrane transport	*MINOS1, CHCHD3, NNT, PAM16, ROMO1, RTN4IP1, SAMM50, SLC25A3, STARD7, TIMM44, TOMM40L*
Mitochondrial RNA processing	*MRPL11, MRPL12, MRPL21, MRPL34, MRPL35, MRPL37, MRPL46, MRPL51, MRPS11, MRPS15, MRPS18A, MRPS33, MRPS34, MRPS35, MRPS36, TBRG4, TRPT1*
Apoptosis associated	*AIFM1, HINT2, MAP3K15, PGAM5, PINK1*
Cellular respiration	*BLOC1S1*
Fatty acid (long chain) beta-oxidation	*PTGES2*
Oxidative phosphorylation related	*ATPIF1, BOLA3, CHCHD10, COA6, ECSIT, IMMT, SIRT5*
Ubiquinone biosynthesis	*COQ5, COQ9*

Genes associated with oxidative phosphorylation (Cluster 17).

Genes encoding proteins involved in oxidative phosphorylation and related pathways from Cluster 17 are shown.

Cluster 10 was enriched for genes with GO terms including “cell cycle” (*p* = 1.14 × 10^−57^), “mitotic cell cycle” (*p* = 1.80 × 10^−44^), and “cell cycle process” (*p* = 5.60 × 10^−42^). These genes cluster together because they have higher expression in tissues with a relatively high proliferative index, such as the small intestine, Peyer’s patches, and the bone marrow.

### GI Tract Gene Expression

Although ruminant species have anatomically equivalent gastrointestinal tracts, we considered that GI tract gene expression may differ due to differences in diet, metabolism, or habitat. To test this hypothesis, we compared gene expression in the GI tract between buffalo and sheep, using gene expression data from the sheep atlas (Clark et al., 2017), which have also been the focus of a separate analysis (Bush et al., 2018). Equivalent datasets from both species were clustered using Graphia Professional with genes expressed in the forestomach and small and large intestines compared between species. Cluster 21 in buffalo contained 131 genes with enriched expression in the forestomach (reticulum, rumen, omasum, abomasum) and tonsils. Co-expression in these tissues was previously observed in the sheep atlas, (Clark et al., 2017) and in earlier studies in sheep (Xiang et al., 2016), and is thought to be due to their similar stratified squamous epithelial layer. The unannotated genes were removed and the remaining 91 buffalo genes compared to cluster 13 of the sheep atlas, which contained 155 (annotated) genes. Approximately, a third of these genes (*n* = 44) were common to both buffalo and sheep clusters. Of the remaining genes, 47 were only present in buffalo cluster 21, and 111 genes were only present in sheep cluster 13. Genes shared by both species include the keratin genes *KRT5*, *KRT15*, *KRT23*, *KRT78*, and *KRT80* and the peptidoglycan recognition protein genes PGLYRP3 and PGLYRP4. Expression of these peptidoglycan receptor proteins has previously been described in the GI tract of other mammals such as mice, humans, and pigs (Liu et al., 2001; Mathur et al., 2004; Lu et al., 2006; Ueda et al., 2011). Genes only detected in buffalo include those encoding the tuft cell marker POU2F3, keratinocyte markers KRT6A and IVL, and the antioxidant enzyme gene *GSR*. Where differences were detected between species, missing genes were present in other clusters of either sheep or buffalo, not detected in the tissues collected for each species, or accounted for by a lack of annotation in one species. A list of these genes along with the relevant sheep or buffalo cluster is found in **Supplementary Table 12**.

Cluster 26 was enriched for genes expressed in the small and large intestines, although with highest expression in the former. Genes expressed in this cluster are enriched for the GO terms “brush border assembly” (*p* = 2.25 × 10^−4^), “regulation of microvillus organization” (*p* = 1.02 × 10^−2^), and “regulation of cell projection size” (*p* = 1.45 × 10^−2^). This cluster includes genes expressed in the crypt-villus axis of the small intestine, such as *BMP5*, *ATOH1*, and *VIL1*, along with the mucin-encoding genes *MUC3A* and *MUC12*.

We also compared levels of expression of the *SLC* gene family between buffalo and sheep. This superfamily comprises 49 gene families across both species, consisting of 342 and 335 genes in buffalo and sheep, respectively. These genes encode membrane-bound transporters, symporters, and antiporters (reviewed in He et al., 2009) and are mainly expressed in the kidney, although there are subsets with tissue-specific expression in the brain, heart, thyroid, and macrophages. The expression of these genes tended to be similar in tissue specificity between buffalo and sheep. There were a few exceptions. *SLC16A1*, reported to be expressed in the cattle rumen (Muller et al., 2002), and expressed at high levels in the sheep forestomach, was barely detectable in any buffalo tissue (TPM < 2). This could be due to an error in the annotation.

### lncRNA Annotation and Expression

**Figure 2:**
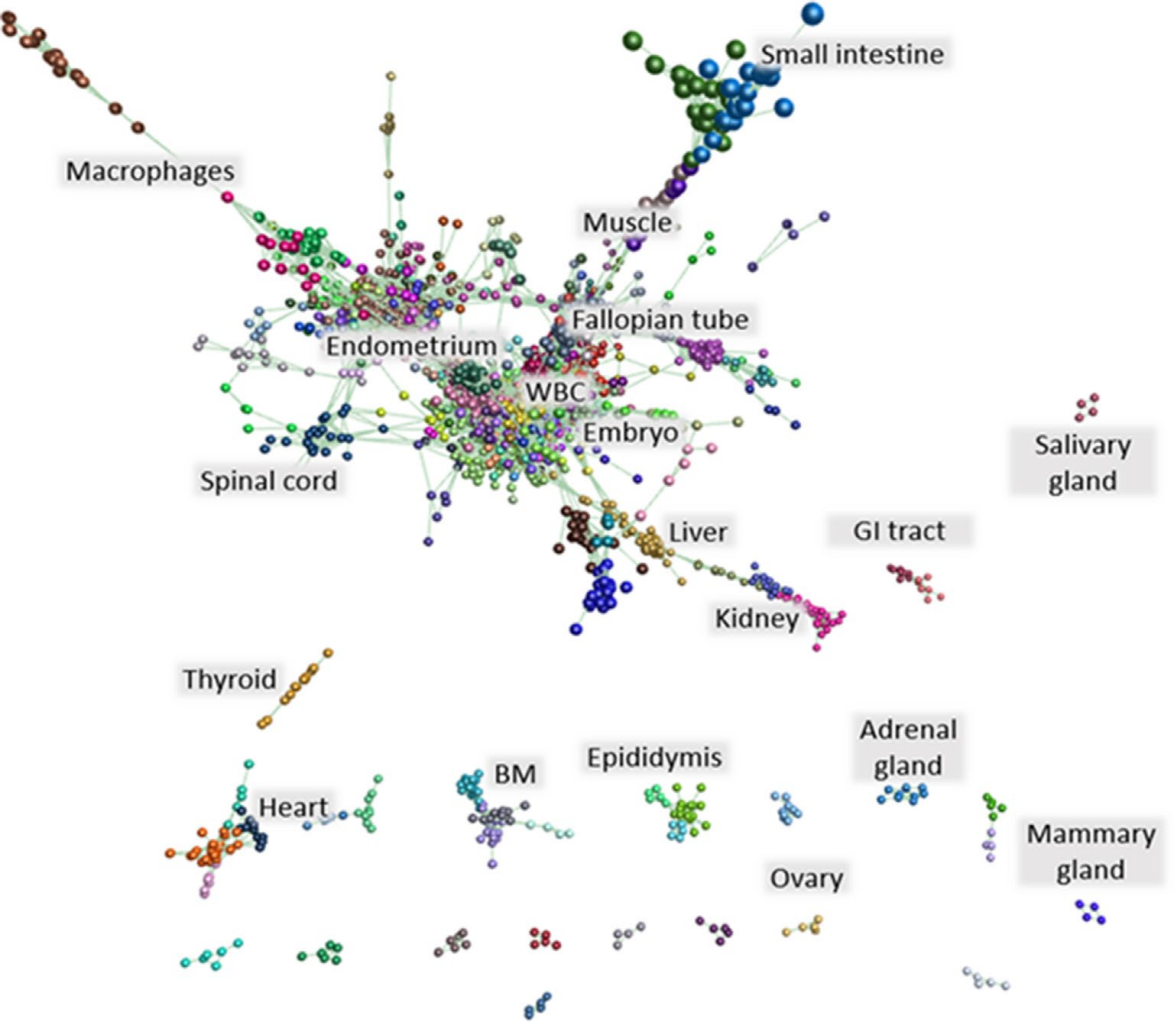
lncRNA network graph. A network graph of annotated lncRNAs was generated applying a correlation threshold of *r* ≥ 0.9. The graph comprised 1,047 nodes and 58,878 edges. Clustering of the graph resulted in 42 clusters of 6 to 394 nodes. These clusters were annotated based on the tissue specificity of co-expressed lncRNAs. **Supplementary Table 13** contains a list of the contents of each cluster.

The detection of long non-coding RNAs (lncRNAs) from large gene expression atlas projects has added a further layer of complexity to the genome and regulation of gene expression. The ENCODE project has annotated approximately 16,000 lncRNAs in the human genome. More recently, lncRNAs have been annotated in livestock and large animal species such as sheep, goat, cattle, pig, and horse (Zhou et al., 2014; Koufariotis et al., 2015; Scott et al., 2017; Bush et al., 2018). We detected 6,756 putative lncRNAs in the buffalo by *de novo* assembly from our buffalo RNA-Seq dataset using methods described previously to generate a catalogue of ruminant lncRNAs in sheep, goat, and cattle (Bush et al., 2018). Expression of these lncRNAs was explored using Graphia Professional. A gene-to-gene correlation matrix was generated and a weighted network graph constructed using a Pearson correlation of *r* ≥ 0.9. The resulting graph contained 1,047 nodes and 58,878 edges. When clustered using MCL, the nodes formed 42 clusters of 6 to 394 nodes. An image of the network graph is shown in **Figure 2**. **Supplementary Table 13** contains a list of the contents of each cluster.

Most of the lncRNA clusters were tissue- or organ system–specific. The largest lncRNA cluster (cluster 1) showed co-expression of lncRNAs in a single buffalo embryo and embryo pool. lncRNA cluster 6, a relatively small cluster containing only 39 lncRNAs, also showed co-expression in the embryo, along with the occipital lobe and longitudinal dorsal muscle. This expression pattern reflects the involvement of lncRNAs in the regulation of gene expression during development (reviewed in Fatica and Bozzoni, 2014). lncRNA cluster 5 contained lncRNAs co-expressed in white blood cells while those in lncRNA cluster 13 were expressed in PBMCs, white blood cells, spleen, and endometrium. lncRNA cluster 7 contained 37 lncRNAs whose co-expression was specific to the fallopian tube. lncRNA cluster 9 was liver-specific, and lncRNA cluster 17 was bone marrow–specific. Each of these clusters of co-expressed lncRNAs merits further investigation into the genes and processes they regulate.

## Conclusion

All of the RNA-Seq data generated in this project have been provided to support annotation of intron-exon boundaries in the new water buffalo genome assembly (Low et al., 2019). The StringTie pipeline was used both to extend the Kallisto index, increasing the number of genes for which abundance can be quantified, and to identify novel lncRNA. However, the data can also provide a framework for the identification of novel splice variants of any gene of interest. For example, we recently analyzed the intron-exon structure of the complex *ADGRE1* locus expressed in macrophages (Waddell et al., 2018). This analysis revealed that ruminants have a duplication of the extracellular domain, and the existence of extensive exon-skipping to encode isoforms that differ in the number of EGF-like calcium-binding domains. The animals used in the atlas are outbred, and the primary data also provide a resource for the analysis of allelic imbalance. Analysis of RNA-Seq data in other species, including cattle (Chamberlain et al., 2015) and humans (Nothnagel et al., 2011; Edsgard et al., 2016), has uncovered extensive allelic variation in gene expression. Finally, we are currently analyzing whole-genome DNA sequences of multiple Indian water buffalo breeds. The intersection of genomic DNA with functional annotation will provide insights into the molecular basis of breed-specific traits.

## Ethics Statement

Ethics approval was obtained from The Roslin Institute’s and the University of Edinburgh’s Protocols and Ethics Committees. All animal work was carried out under the regulations of the Animals (Scientific Procedures) Act 1986.

## Author Contributions

RY, LL, SG, SK, AA and DH contributed conception and design of the study; SB performed data analysis and curation; RY, LL, AJ, SS, SJ, VD, ZL, DI, KS, JW and DH collected the samples. LL, RY, AJ, SS, SJ and VD performed the experiments. RY, LL, SB and DH analysed the results. DH, AA, SK and SG secured funding and supervised the project. RY wrote the first draft of the manuscript; LL, SB and DH wrote sections of the manuscript. All authors contributed to manuscript revision, read and approved the submitted version.

## Funding

This work was supported by a joint Biotechnology and Biological Sciences Research Council (BBSRC) (http://www.bbsrc.ac.uk) and Indian Department of Biotechnology Grant BB/L004623/1 (‘Transcriptome Analysis in Indian Buffalo and the Genetics of Innate Immunity’), also BBSRC Institute Strategic Program Grants: BBS/E/D/20211550 (‘Farm Animal Genomics’), BBS/E/D/20211552 (‘Transcriptomes, Networks and Systems’) and BB/P013732/1 (‘Blueprints for Healthy Animals’). Edinburgh Genomics is partly supported through core grants from BBSRC (BB/J004243/1), NERC (http://www.nerc.ac.uk) (R8/H10/56) and the Medical Research Council (MRC) (https://www.mrc.ac.uk) (MR/K001744/1). KMS and DAH receive funding form the Mater Foundation, Brisbane, Australia.

## Conflict of Interest Statement

The authors declare that the research was conducted in the absence of any commercial or financial relationships that could be construed as a potential conflict of interest.

## Footnotes

1
http://www.fao.org/faostat

2
www.kajeka.com/graphia-professional

3
http://www.biogps.org

4
http://www.pantherdb.org

5
https://www.ebi.ac.uk/biosamples/samples

6
https://www.ebi.ac.uk/ena

7
http://ftp.faang.ebi.ac.uk/ftp/protocols
